# Characteristics of Users of a Digital Hypnotherapy Intervention for Hot Flashes: Retrospective Study

**DOI:** 10.2196/53555

**Published:** 2024-03-14

**Authors:** Morgan Snyder, Gary R Elkins

**Affiliations:** 1 Department of Psychology and Neuroscience Baylor University Waco, TX United States

**Keywords:** hypnotherapy, hot flashes, smartphone app, mHealth, mobile health, app, apps, applications, hypnosis, menopause, menopausal, gynecology, usage, women's health, user, users, demographics, demographic, characteristic, characteristics, mental health, alternative, complementary, mind body, hypnotism

## Abstract

**Background:**

Hot flashes are associated with a lower quality of life and sleep disturbances. Given the many consequences of hot flashes, it is important to find treatments to reduce them. Hypnotherapy, the use of hypnosis for a medical disorder or concern, has been shown in clinical trials to be effective in reducing hot flashes, but it is not routinely used in clinical practice. One solution to close this implementation gap is to administer hypnotherapy for hot flashes via a smartphone app. Evia is a smartphone app that delivers hypnotherapy for hot flashes. Evia has made hypnotherapy more widely accessible for women who are experiencing hot flashes; however, the app has yet to undergo empirical testing. Additionally, research on user characteristics is lacking.

**Objective:**

This study aims to (1) determine the average age, stage of menopause, and length of menopause symptoms for users of the Evia app; (2) determine the characteristics of hot flashes and night sweats for users of the Evia app; (3) determine the self-reported sleep quality of users of the Evia app; (4) determine the self-reported mental health of users of the Evia app; and (5) determine the relationship between hot flash frequency and anxiety and depression for users of the Evia app.

**Methods:**

This study analyzed data collected from participants who have downloaded the Evia app. Data were collected at 1 time point from a self-report questionnaire that assessed the demographic and clinical characteristics of users. The questionnaire was given to users when they downloaded the Evia app. Users of the Evia app fill out a questionnaire upon enrolling in the program and prior to beginning the intervention. This included 9764 users.

**Results:**

Results showed that the mean age of users was 49.31 years. A total of 41.6% (1942/4665) of users reported experiencing 5 or more hot flashes per day, while 51.2% (1473/2877) of users reported having difficulty falling asleep each night and 47.7% (1253/2626) of users reported their sleep quality to be terrible. In addition, 38.4% (1104/2877) of users reported that they often feel anxious or depressed. There was a small, significant, and negative correlation between hot flash frequency and self-report frequency of anxiety and depression (*r*=–0.09).

**Conclusions:**

This study showed that the average age of app users is in line with the median age of natural menopause. A large percentage of users reported experiencing 5 or more hot flashes per day, reported difficulties with sleep, and reported experiencing depression and anxiety. These findings are in line with previous studies that assessed hot flash frequency and the consequences of hot flashes. This was the first study to report on the characteristics of users of the Evia app. Results will be used to optimize the hypnotherapy program delivered via the Evia app.

## Introduction

Hot flashes are common during menopause, which is marked by 12 months of amenorrhea or absence of the menstrual period. It is estimated that approximately 75% of women experience hot flashes during menopause [1]. During the transition into menopause, hormone levels fluctuate, and this leads to many physical changes and symptoms, with hot flashes being known as the “hallmark” symptom of menopause. Hot flashes are distressing for women who experience them, and they are associated with a lower quality of life [2] and psychological well-being [3]. Hot flashes are also associated with sleep disturbances [4-6]. Given the many consequences of hot flashes, it is important to find treatments to reduce them.

The most effective treatment for reducing hot flashes is hormone therapy; however, this has been shown to increase the risk of breast cancer and its use is contraindicated in many situations such as in breast cancer survivors [7]. Other treatments for hot flashes such as selective serotonin reuptake inhibitors, selective norepinephrine reuptake inhibitors, clonidine, gabapentin, cognitive behavioral therapy, and mindfulness-based stress reduction have not been found to be as effective as hormone therapy, and they often include side effects that make their long-term use undesirable [8-13]. Therefore, effective and safe nonpharmacological treatment options are needed to relieve hot flashes.

Hypnotherapy, the use of hypnosis for a medical disorder or concern, has been shown to be effective in reducing hot flashes [14,15]. In a clinical trial that examined the efficacy of hypnotherapy for reducing hot flashes among breast cancer survivors, it was found that hypnotherapy reduced hot flashes by approximately 68% from baseline to intervention end point and this was significantly greater than controls [14]. In another clinical trial that examined the efficacy of hypnotherapy for reducing hot flashes among postmenopausal women, hot flashes were reduced by approximately 80% from baseline to week 12 and these reductions were significantly greater than the structured attention control condition [15].

Although hypnotherapy has been shown to be effective in clinical trials, it is not routinely used in clinical practice. Very few clinicians are trained to administer hypnotherapy for hot flashes. One solution to close this implementation gap is to administer hypnotherapy for hot flashes via a smartphone app. Evia is a smartphone app that delivers hypnotherapy for hot flashes. It is based on a hypnotherapy protocol that has been rigorously tested in randomized clinical trials [14,15] and was developed in consultation with an expert in the field of hypnotherapy for hot flashes. Evia includes a 5-week hypnotherapy intervention with daily tasks that include listening to the audio-recorded hypnotherapy session, tracking hot flashes, and psychoeducational readings. Evia has made hypnotherapy more widely accessible for women who are experiencing hot flashes; however, the app has yet to undergo empirical testing. Additionally, research on user characteristics is lacking. This is the first study to report the characteristics of users of Evia. The information gathered from this study will allow for the Evia app to be tailored and optimized toward its users. This study will also tell us whether individuals who seek relief from hot flashes via a smartphone app are different from those who seek treatment in a clinical setting or a clinical trial, and will allow us to better understand who is downloading and engaging with the digital hypnotherapy intervention. Some previous research has shown that smartphone app users are generally younger [16], and this study will explore whether users of Evia are younger than women who seek treatment in a clinical setting or clinical trial. Additionally, the study will explore how app users may differ in the length of time they have experienced menopause symptoms or symptom severity. In addition, this study is a first step to conducting further research on the Evia app, including clinical trials to evaluate efficacy.

This study analyzed data collected from participants who have downloaded the Evia app. These data were collected by Mindset Health, the developers of the Evia app. Users of the Evia app fill out a questionnaire upon enrolling in the program and prior to beginning the intervention. These data will be analyzed to achieve the study’s aims. This study aims to (1) determine the average age, stage of menopause, and length of menopause symptoms for users of the Evia app; (2) determine the characteristics of hot flashes and night sweats for users of the Evia app; (3) determine the self-reported sleep quality of users of the Evia app; (4) determine the self-reported mental health of users of the Evia app; and (5) determine the relationship between hot flash frequency and anxiety and depression for users of the Evia app.

## Methods

### Overview

Data were collected at 1 time point from a self-report questionnaire that was given to users when they downloaded the Evia app between October 5, 2021, and July 8, 2022. Users filled out the self-report questionnaire directly on the Evia app. These data were collected prior to the user beginning the intervention and were analyzed retrospectively.

### Participants

Participants included individuals who downloaded the Evia app between October 5, 2021, and July 08, 2022. Participants are users of the Evia app and were recruited via Facebook advertisements and the app. Any person who downloaded the Evia app during this time period was prompted to fill out a questionnaire with demographic and clinical information prior to beginning the intervention. This included 9764 participants.

### Measures

#### Aim 1

Participants were asked to fill in their age. Participants were asked, “How would you classify your stage of menopause?” Response options included “perimenopause,” “menopause,” “postmenopausal,” “I’m not menopausal,” and “I’m not sure.” To determine the amount of time that users have been experiencing menopause symptoms, participants were asked, “How long have you been experiencing menopause symptoms?” Response options included “0-6 months,” “6-12 months,” “1-2 years,” “2-3 years,” “3-5 years,” “5-10 years,” and “10+ years.”

#### Aim 2

Participants were asked, “How many hot flashes do you experience each day?” Response options ranged from “0” to “5+.” To assess the severity of hot flashes that users are experiencing, participants were asked, “How severe are your hot flashes?” Response options included “very mild”, “mild”, “moderate”, “severe”, and “very severe.” To assess the characteristics of users’ hot flashes, they were asked, “What do your hot flashes feel like?” Response options included “sudden feeling of warmth,” “perspiration,” “flushed appearance,” “rapid heartbeat,” “anxiety/an aura,” “dizziness/weakness,” “nausea,” and “other.” Users were able to select multiple responses for this item. To assess the number of night sweats that users experience, they were asked, “How many night sweats do you experience each night, on average?” Response options ranged from “0” to “5+.”

#### Aim 3

Users were asked to report sleep difficulty with 1 self-report item that reads, “Many women also struggle with sleep during menopause. Do you find it difficult to fall asleep each night?” Response options include “no,” “a little bit,” and “yes.” Users were also asked to rate their sleep quality with 1 self-report question that read, “How would you rate the quality of your sleep?” Response options included “terrible,” “fair,” “good,” and “excellent.”

#### Aim 4

Users were asked 1 self-report question that read, “Some women also struggle with mental health during menopause. How often do you feel anxious or depressed?” Response options included “never,” “sometimes,” “often,” and “constantly.”

#### Aim 5

Users were asked, “How many hot flashes do you experience each day?” Response options ranged from “0” to “5+.” Users were asked, “Some women also struggle with mental health during menopause. How often do you feel anxious or depressed?” Response options included “never,” “sometimes,” “often,” and “constantly.”

### Ethical Considerations

This study was reviewed by the institutional review board at Baylor University and was considered to be exempt. The institutional review board determined the study to be secondary research for which consent is not required. The data analyzed were deidentified.

### Data Analyses

Descriptive statistics (means, SDs, and frequencies) were calculated for the variables of interest. For aim 5, which was to determine the relationship between hot flash frequency and self-report frequency of anxiety and depression, a Pearson correlation analysis was used.

## Results

### Aim 1: Age, Stage of Menopause, and Length of Menopause Symptoms

The mean age of users was 49.31 (SD 6.693) years. Out of 9103 valid responses, 0.5% (n=49) of users reported being not menopausal, 31.2% (n=2837) of users were perimenopausal, 14.2% (n=1293) of users were menopausal, 13.1% (n=1188) of users were postmenopausal, 7.6% (n=688) of users were uncertain, and 33.5% (n=3048) of users reported unknown (see Table 1). Out of 3127 valid responses reporting length of experiencing menopause symptoms, 20.4% (n=639) of users reported 0-6 months, 20.9% (n=655) of users reported 6-12 months, 20% (n=624) of users reported 1-2 years, 13.5% (n=421) of users reported 2-3 years, 11.3% (n=354) of users reported 3-5 years, 9.3% (n=290) of users reported 5-10 years, and 4.6% (n=144) of users reported 10+ years (see Table 1).

**Table 1:** Stage of menopause and length of menopause symptoms for Evia users. This table lists the frequencies and percentages of Evia app users who are in each stage of menopause and their length of menopause symptoms. These data were collected from Evia app users who downloaded the Evia app. Data were collected via a questionnaire given on the Evia app to users prior to beginning the intervention and were analyzed retrospectively. These data were collected between October 5, 2021, and July 8, 2022. The Evia app includes a hypnotherapy intervention to help reduce hot flashes.

Variable		Frequency, n (%)
**Stage of menopause (n=9103)**		
	Not menopausal	49 (0.5)
	Perimenopausal	2837 (31.2)
	Menopausal	1293 (14.2)
	Postmenopausal	1188 (13.1)
	Uncertain	688 (7.6)
	Unknown	3048 (33.5)
**Length of menopause symptoms (n=3127)**		
	0-6 months	639 (20.4)
	6-12 months	655 (20.9)
	1-2 years	624 (20)
	2-3 years	421 (13.5)
	3-5 years	354 (11.3)
	5-10 years	290 (9.3)
	10+ years	144 (4.6)

### Aim 2: Hot Flash Characteristics

Out of 4665 valid responses, 13.5% (n=630) of users reported experiencing 0 hot flashes per day, 9.4% (n=437) of users reported experiencing 1 hot flash per day, 13% (n=607) of users reported experiencing 2 hot flashes per day, 12.8% (n=595) of users reported experiencing 3 hot flashes per day, 9.7% (n=454) of users reported experiencing 4 hot flashes per day, and 41.6% (n=1942) of users reported experiencing 5+ hot flashes per day (see Table 2). Out of 4183 valid responses, 1.9% (n=80) of users reported that their hot flashes were very mild, 30.4% (n=1273) of users reported that their hot flashes were mild, 40.9% (n=1710) of users reported that their hot flashes were moderate, 20.3% (n=851) of users reported that their hot flashes were severe, and 6.4% (n=269) of users reported that their hot flashes were very severe (see Table 2). Out of 4180 valid responses, 83% (n=3469) of users reported that their hot flashes felt like a sudden feeling of warmth, 59.2% (n=2474) of users reported perspiration, 47.5% (n=1987) of users reported a flushed appearance, 34.5% (n=1442) of users reported rapid heartbeat, 32.7% (n=1367) of users reported anxiety or an aura, 24.5% (n=1023) of users reported dizziness or weakness, and 16.4% (n=686) of users reported nausea (see Table 2). Out of 321 valid responses, 13.4% (n=43) of users reported experiencing 0 night sweats, 25.5% (n=82) of users reported experiencing 1 night sweat, 23.1% (n=74) of users reported experiencing 2 night sweats, 21.8% (n=70) of users reported experiencing 3 night sweats, and 16.2% (n=52) of users reported experiencing 4 night sweats (see Table 2).

**Table 2:** Number, severity, and characteristics of hot flashes for Evia users. This table lists the frequencies and percentages for Evia app users’ number of hot flashes, severity of hot flashes, hot flash characteristics, and number of night sweats. These data were collected from Evia app users who downloaded the Evia app. Data were collected via a questionnaire given on the Evia app to users prior to beginning the intervention and were analyzed retrospectively. These data were collected between October 5, 2021, and July 8, 2022. The Evia app includes a hypnotherapy intervention to help reduce hot flashes.

Variable			Frequency, n (%)
**Number of hot flashes (n=4665)**			
	0	630 (13.5)	
	1	437 (9.4)	
	2	607 (13)	
	3	595 (12.8)	
	4	454 (9.7)	
	5+	1942 (41.6)	
**Severity of hot flashes (n=4183)**			
	Very mild	80 (1.9)	
	Mild	1273 (30.4)	
	Moderate	1710 (40.9)	
	Severe	851 (20.3)	
	Very severe	269 (6.4)	
**Hot flashes feel like (n=4180)**			
	Sudden feeling of warmth	3469 (83)	
	Perspiration	2474 (59.2)	
	Flushed appearance	1987 (47.5)	
	Rapid heartbeat	1442 (34.5)	
	Anxiety or an aura	1367 (32.7)	
	Dizziness or weakness	1023 (24.5)	
	Nausea	686 (16.4)	
	Other	307 (7.3)	
**Number of night sweats (n=321)**			
	0	43 (13.4)	
	1	82 (25.5)	
	2	74 (23.1)	
	3	70 (21.8)	
	4	52 (16.2)	

### Aim 3: Sleep

Out of 2877 valid responses, results demonstrated that 18% (n=519) of users reported no to having difficulty falling asleep each night, 30.8% (n=885) of users reported a little bit of difficulty falling asleep each night, and 51.2% (n=1473) of users reported yes to having difficulty falling asleep each night. Out of 2626 valid responses, results also demonstrated that 47.7% (n=1253) of users reported their sleep quality to be terrible, 44% (n=1155) of users reported their sleep quality to be fair, 7.8% (n=206) of users reported their sleep quality to be good, and 0.5% (n=12) of users reported their sleep quality to be excellent.

### Aim 4: Mental Health

Out of 2877 valid responses, 5.9% (n=169) of users reported that they never feel anxious or depressed, 39.6% (n=1139) of users reported that they sometimes feel anxious or depressed, 38.4% (n=1104) of users reported that they often feel anxious or depressed, and 16.2% (n=465) of users reported that they constantly feel anxious or depressed.

### Aim 5: Relationship Between Hot Flash Frequency and Anxiety and Depression

There was a small, significant, and negative correlation between hot flash frequency and self-report frequency of anxiety and depression (*r*(2875) = –0.09, *P*<.001).

## Discussion

### Principal Findings

Results demonstrated that the average age of app users (49.31 years) is in line with the median age of the beginning of perimenopause (47.5 years) and the median age of natural menopause (51.3) [17]. Although previous studies have shown that, overall, app users are generally younger [16], users of Evia are the age we would expect based on the age of menopause onset and symptoms emerging. Additionally, a large percentage of users (2837/9103, 31.2%) reported being in perimenopause, and this is oftentimes when menopause symptoms, including hot flashes, begin. An unexpected finding in this study was that a large percentage of users did not know their stage of menopause (3048/9103, 33.5%). This shows that users of Evia may not be aware of the various stages of menopause and what the menopause transition entails. This suggests that it may be beneficial for the Evia app to include some psychoeducational components about the menopause transition and stages of menopause. These psychoeducational components may help users of Evia better understand their menopause transition and experiences. Many users reported experiencing menopause symptoms for 0-6 months (639/3127, 20.4%), 6-12 months (655/3127, 20.9%), or 1-2 years (624/3127, 20%) with users less commonly reporting experiencing menopause symptoms for 5-10 years (290/3127, 9.3%) or 10+ years (144/3127, 4.6%). These results demonstrate that users are starting to use Evia toward the beginning of menopause symptoms. It is possible that those who have been experiencing symptoms for longer have learned how to cope with or treat their symptoms.

The largest percentage of users reported experiencing 5 or more hot flashes per day and reported that their hot flashes were moderate intensity. This finding is in line with previous studies that assessed hot flash frequency at baseline [15]. In a clinical trial by Elkins et al [15], participants experienced approximately 10 hot flashes per day on average at baseline. This study showed that, on average, Evia app users experience 5 or more hot flashes per day, which is in line with participants in previous clinical trials.

In line with previous studies regarding hot flashes and sleep, a majority of users (1473/2877, 51.2%) reported difficulty falling asleep each night and reported their sleep quality to be terrible (1253/2626, 47.7%) or fair (1155/2626, 44%). Previous studies have shown that menopausal women generally have poorer sleep than those not in menopause [18]. In addition, previous studies have also shown that hot flashes are associated with worse sleep outcomes [4-6]. Therefore, the finding that Evia users are reporting sleep problems is in line with previous research. Hypnotherapy interventions have been shown to improve sleep in menopausal women [15,19]. Evia includes components to address sleep problems and this study suggests that those components should be kept or additional components should be added in order to help users who are reporting difficulty falling asleep and poor sleep quality.

A majority of users reported that they sometimes (1139/2877, 39.6%) or often (1104/2877, 38.4%) feel anxious or depressed. This is also in line with previous research which has shown associations between hot flashes and symptoms of anxiety or depression [20,21]. However, this study found that there was a small, negative correlation between hot flash frequency and frequency of anxiety and depression, and this did not support the hypothesis that there would be a positive correlation between hot flash frequency and frequency of anxiety and depression. As mentioned above, previous research has found positive associations between hot flashes and symptoms of anxiety and depression; therefore, this finding is not in line with previous research. The correlation of –0.09 is also very small and near zero. It is likely only significant due to the large sample size. There are a couple of reasons that this finding may have occurred. First, users were not able to report the true number of hot flashes that they may have been experiencing and were limited to selecting 0, 1, 2, 3, 4, or 5+. Therefore, we measured a limited range of measurement of number of hot flashes. If users were able to report the exact number of hot flashes they were experiencing, there may have been more variation in this variable, and this may have affected the correlation. Second, the measurement of the frequency of anxiety or depression symptoms with a single item is not optimal. If a validated scale that measures depression or anxiety had been used, the measurement of this variable would have been improved. There may have been greater variability in this variable and the correlation may have been different. In addition, validated scales generally ask about the frequency of specific symptoms experienced whereas the single-item measure in this study asked solely about how often one was feeling anxious or depressed. Therefore, by using the single-item scale, the construct may have not been fully captured.

### Limitations

This study has several limitations that should be addressed. First, several characteristics of users remain unknown and were not addressed by this study. For example, information about geographic location and race or ethnicity was not reported. The study was not able to describe several important demographic factors of the sample, including race or ethnicity and education level.

There were also limitations regarding how variables in the study were measured. For example, sleep quality and frequency of anxiety and depression symptoms were measured using single items. There are limitations to using single-item scales, including that they may not accurately or fully capture the construct. In addition, the items may have been worded in a suggestive manner. For example, “Many women also struggle with sleep during menopause. Do you find it difficult to fall asleep each night?” By starting the item with, “Many women also struggle with sleep during menopause...” it may be suggested for the individual to respond in a certain way. By identifying these limitations of measurement, this study will help to inform the optimization of data collection and measurement with the Evia app.

### Future Directions

The results of this study will inform the optimization of the hypnotherapy intervention delivered via the Evia app. The results of this study will also inform the optimization of data collection via the Evia app. Once the Evia app is optimized, further research is warranted. Future studies should first examine the feasibility of the Evia app in a randomized controlled trial design. Then, future studies should examine the efficacy of the Evia app in comparison to a control group or even a face-to-face delivery.

### Conclusions

This was the first study to report on the characteristics of users of the Evia app, which delivers hypnotherapy for hot flashes. Results showed that the average age of app users is in line with the average age of menopause onset. Results also showed that the largest percentage of users reported experiencing 5 or more hot flashes per day and reported that their hot flashes were moderate intensity. In line with previous research regarding hot flashes and sleep, a majority of users reported difficulty falling asleep each night and reported their sleep quality to be terrible or fair. In addition, a majority of users reported that they sometimes or often feel anxious or depressed. The results of this study will help to inform the optimization and tailoring of the hypnotherapy intervention delivered via the Evia app.

## Data Availability

The data set analyzed during this study is not publicly available due to confidentiality but is available from the corresponding author upon reasonable request.
